# Novel perspective of therapeutic modules to overcome motor and nonmotor symptoms in Parkinson's disease

**DOI:** 10.3934/Neuroscience.2024020

**Published:** 2024-09-06

**Authors:** Anmol Kumar, Ajay Kumar Gupta, Prashant Kumar Singh

**Affiliations:** 1 School of Pharmaceutical Science (formerly University Institute of Pharmacy), Chhatrapati Shahu Ji Maharaj University (formerly Kanpur University), Kanpur 208024, India; 2 Institute of Pharmaceutical Sciences, University of Lucknow, India

**Keywords:** Parkinson's disease (PD), motor and non-motor symptoms, dopamine precursor, deep brain stimulation (DBS), ablative surgery

## Abstract

Parkinson's disease (PD) is a neurodegenerative disorder that involves the loss of dopaminergic neurons, which leads to motor and non-motor symptoms that have a significant impact. The pathophysiology of PD is complex and involves environmental and genetic factors that contribute to alpha-synuclein aggregation, mitochondrial dysfunction, oxidative stress, and neuroinflammation. The current treatments of PD primarily focus on symptom management and have limitations in addressing disease progression and non-motor symptoms. Epidemiological data indicates a rise in PD cases worldwide, which highlights the need for effective treatments. Pathophysiological insights point out the involvement of various factors in PD progression, such as dopamine dysregulation, genetic mutations, oxidative stress, mitochondrial damage, alpha-synuclein aggregation, and neuroinflammation. Although current treatments, which include dopamine precursors, monoamine oxidase (MAO) inhibitors, and non-dopaminergic drugs, can alleviate motor symptoms, they are not effective in preventing disease progression or managing non-motor symptoms. Additionally, they can lead to adverse effects and become less effective over time. Novel therapeutic approaches, including cell-based therapies, gene therapies, targeted drug delivery therapies, and magnetic field therapies, are promising in improving symptom management and providing personalized treatment. Additionally, emerging therapies that target alpha-synuclein aggregation, mitochondrial dysfunction, and neuroinflammation may have potential disease-modifying effects. To sum up, for dealing with the multiple aspects of PD, there is a great need to come up with new and creative therapeutic approaches that not only relieve symptoms, but also prevent the progression of disease and non-motor symptoms. The progress made in comprehending the underlying mechanisms of PD provides optimism for developing successful treatments that can enhance the outcomes and quality of life.

## 1. Introduction

Parkinson's disease (PD) is the second most common neurodegenerative disorder after Alzheimer's disease, which harms millions of people worldwide. The development of Lewy body aggregates in PD is associated with a progressive loss of dopaminergic neurons in the substantia nigra (SN) region of the midbrain and the striatal nerve fiber projections (STR). PD is a complex movement disorder in which the motor symptoms, such as bradykinesia, tremors, and postural stiffness, are brought on by the loss of dopaminergic neurons. Non-motor symptoms include depression, anxiety, sleep disorders, and gastrointestinal problems, among other things. There are idiopathic and hereditary variants of the illness; however, idiopathic PD accounts for 90–95% of cases[1],[2]. In 1817, the London surgeon James Parkinson initially characterized PD in his influential work “An Essay on the Shaking Palsy”. PD was once known as “paralysis agitans” or “shaking palsy”. Parkinson described the hallmark signs of the illness in this essay, which included an involuntary tremulous motion, decreased muscle power, a tendency to bend the trunk forward, and a shuffling stride, all without affecting the senses or the brain [3],[4]. PD is becoming more prevalent globally, which raises serious concerns for public health. In those over 60, the estimated prevalence of PD is 1%. The main biochemical changes in PD include a reduction in tyrosine hydroxylase (TH), which is the enzyme responsible for dopamine synthesis, and a decrease in dopamine levels, both of which involve anatomical pathology. Dopamine is highly susceptible to oxidative stress and auto-oxidizes, thus producing free radicals. The pathophysiology of PD is complex and involves various factors, such as protein aggregation, endoplasmic reticulum (ER) stress, disturbances in calcium metabolism, oxidative & nitrosative stress, mitochondrial impairment, and peroxisomal and endosome-lysosomal dysfunction. Protein misfolding and aggregation are specifically implicated in PD pathology, as evidenced by Lewy bodies that contain aggregates of α-synuclein, molecular chaperones, and ubiquitin, which represent impaired function of the ER and protein degradation mechanisms [1].

Major symptoms of PD are characterized by a variety of motor and non-motor symptoms that have a significant impact on the quality of life of those affected. The movement-related motor symptoms include tremors, Bradykinesia, rigidity, and postural instability, all of which are well-known indicators of the disease and can vary in severity and progression [5]. Meanwhile, the non-motor symptoms, which are often neglected but are equally important, cover a wide range of issues such as depression, anxiety, cognitive impairment, constipation, pain cramps, loss of smell, excessive daytime sleepiness, and rapid eye movement (REM) sleep behavior disorder. It's worth noting that non-motor symptoms can appear years before any motor symptoms, which underscores the complexity and early subtleties of PD. It is crucial to acknowledge and treat non-motor symptoms alongside motor symptoms in individuals with PD, as non-motor symptoms can have a profound impact on their quality of life. To ensure that PD patients receive comprehensive care and experience an overall improvement in their well-being, it is imperative to manage and comprehend both their motor and non-motor symptoms [6],[7].

According to the World Health Organization, the occurrence of PD on a global level has increased two times over the past 25 years, making it a serious health concern worldwide. The current estimates predict that approximately 8.5 million persons suffer from PD. In addition to having a substantial impact on the individual, persons with PD are estimated to have 5.8 million disability-adjusted life years (DALYs), which is an 81% increase since 2000. PD not only affects the patient, but also puts a significant burden on healthcare systems [8]. Additionally, PD has been connected to over 329,000 deaths worldwide, which is an increase of more than 100% since 2000. The Global Burden of Disease Study provides a better understanding of global health issues. The study's results highlighted the growing prevalence of PD by revealing that 1.02 million new cases of the condition were recently diagnosed. These figures illustrated the incidence of and added to the total prevalence of PD, and brought up issues in the public health domain [9]. According to a recent study, 0.58 million persons in India were diagnosed with PD; this number may rise in the next decades [10]. The condition of PD is brought on by a complicated interaction between environmental and genetic variables that results in the death and destruction of dopaminergic neurons in the substantia nigra area of the brain. destruction of dopaminergic neurons in the SN area of the brain. Although the precise origins are unknown, research points to a possible mix of genetic alterations and environmental exposures as the culprit. About 10–15% of Parkinson's cases are genetically related, and the disease has been connected to many gene alterations [11]. Although inherited PD is uncommon, parents might pass on defective genes to their offspring. Additionally, there is evidence linking environmental variables such as head injuries, traffic pollution, herbicides, and pesticides to a higher incidence of PD. Nevertheless, there is conflicting data relating certain environmental variables to PD [12],[13].

However, the involvement of major autosomal dominant and recessive genes involved in PD pathology are SNCA, PARK3, UCHL1, LRRK2, PINKI, PARK7, and FBXO7. A mutation in the SNCA gene causes the aggregation of a-synuclein, which is called a Lewy body, and is considered a pathological characteristic of PD. The diagnostic tools available to identify PD are scoring (UPDRS and H&Y) and imaging techniques, which can only be utilized after the appearance of symptoms. Since the disease-related symptoms (motor impairment) appear after the death of 80% of the dopaminergic neurons, a disease diagnosis is a major constraint in the treatment of patients [14]. The occurrence and prevalence of PD considerably vary across different ethnicities and geographical locations. North America and Europe have higher documented rates of PD compared to Asia and Africa. PD is becoming a global health concern because the incidence rate of PD is highly significant and affects the healthy life of an individual and is rapidly increasing in its occurrence. Most of the current therapeutic approaches focus on the symptomatic relief and restoration of dopamine levels and the adjustment of the neurotransmitter activity or function. Despite their efficacy in managing motor symptoms at the onset, these drugs do not tackle the fundamental neurodegenerative processes, which consequently results in the advancement of the disease over a period. Current therapies have certain limitations which highlight the immediate requirement for new therapeutic strategies. The objective is to go beyond managing the symptoms to modify the disease and provide neuroprotection. The progress made in comprehending the molecular mechanisms of PD has presented opportunities for specific therapies that can potentially decelerate or even stop the advancement of the disease. In this review, we focus on and describe the current therapeutics and novel therapies to overcome this global medical health concern [15].

## 2. Pathophysiology of PD and therapeutic target

The pathophysiological landscape of PD is primarily driven by two processes: the pathological aggregation of alpha-synuclein (α-syn), which causes Lewy bodies to develop, and misfolded α-syn's cytotoxic actions to damage dopaminergic neurons in the SN. The development of PD symptoms is the consequent disturbance of normal brain transmission. While the sequence of events leading to neuronal death is becoming more and more recognized, the original cause of these events is still unknown [16],[17].

### 2.1. Role of Dopamine

In the brain, tyrosine undergoes a conversion into dopamine, which is an important neuromodulator that belongs to the catecholamine family of neurotransmitters. Phenylalanine hydroxylase, which is present in the liver, transforms phenylalanine into tyrosine, which is then transported to the brain. After dopamine is produced, the vesicular monoamine transporter 2 (VMAT2) prepares it for release into the synaptic cleft by storing it in synaptic vesicles. The function of dopamine occurs through two primary receptor families, namely D1, which includes subtypes 1 and 5, and D2, which includes subtypes 2, 3, and 4. These receptors are distributed across different regions of the brain and are linked to adenyl cyclase, which modulates their function based on their location [18].

Two enzymes, monoamine oxidase (MAO) and catechol-O-methyl transferase (COMT), metabolize dopamine post-synaptic activity. The expression of the MAO-A isoform in catecholaminergic neurons and the MAO-B isoform in astrocytes facilitates the breakdown of dopamine. COMT primarily resides within glial cells. MAO production and DOPA levels in the brain are inversely correlated. Research is being done on the dopaminergic system and dopamine metabolism as potential PD treatments [19].

Despite tremendous progress, no single illness-modifying medication or cure exists for PD. In addition, due to the advances in the disease, the current main objective of therapy is to lessen the motor and non-motor symptoms. Numerous therapies are available, and continuing research examines potential novel therapeutic avenues.

A recent study provided information about the dynamic regulation of the dopamine transporter (DAT) in neurotransmission and homeostasis, and emphasized the DAT's function in dopamine signaling regulation and its significance in PD pathogenesis. Furthermore, new imaging methods have demonstrated how dopamine affects the brain's neuronal activity across the body, thus highlighting its effects on the motivation, behavior reinforcement, and movement. Understanding these pathways is crucial to develop customized therapies that may alter the course of PD and to improve the results for patients [20],[21].

### 2.2. Role of genetic mutation

Recent developments in the study of PD has increased attention to the intricate genetic makeup that influences the illness. Five to ten percent of PD conditions have been connected to more than 20 genes and many risk-associated variations. Notably, PD pathophysiology is closely associated with mutations in genes such as SNCA, LRRK2, PRKN, PINK1, and GBA. The SNCA gene, which is responsible for encoding the α-Synuclein protein, has been associated with dopaminergic neuronal degeneration [22]. Mutations in the SN pars compacta, such as A53T, A30P, and E46K in SNCA, cause Lewy body aggregation and neuronal death. These α-Synuclein oligomers can activate the TLR2 signaling pathway, which in turn can cause neuroinflammation and microglial activation, as well as the expulsion of mediators of inflammation, including reactive species and the important gene, LRRK2, which is frequently linked to both sporadic and familial cases of PD. It affects autophagy and membrane transport as well as lysosomal biology. PD is linked to mitochondrial malfunction due to mutations in the parkin protein gene, which generates an E3 ubiquitin ligase necessary for eliminating damaged mitochondria by autophagy. Additionally, mutations in the PINK1 gene impact mitochondrial upkeep [23],[24].

In particular, L444P and N370S mutations are frequent genetic risk factors for PD that occasionally occur (sporadic PD and frequently cause rapid cognitive deterioration. These mutations are linked to lysosomal dysfunction and protein aggregation. One genetic mutation was recently identified and sheds light on the causes of PD at the molecular level and may guide future therapeutic approaches. Furthermore, a prospective avenue for therapeutic interventions has been revealed with the identification of a protective genetic mutation in the mitochondrial microprotein SHLP2. Furthermore, an integrated genomics approach has identified 50 novel candidate genes that alter PD pathogenesis; additionally, information about the function of the CHCHD2 gene mutation in familial PD has been established [25],[26].

### 2.3. Oxidative stress and Mitochondrial damage

PD etiology is becoming understood to be largely influenced by mitochondrial dysfunction. Recent research has reinforced the knowledge that perturbations in mitochondrial dynamics and bioenergetics are critical to PD and can result in calcium imbalance, oxidative stress, decreased ATP synthesis, and neuronal damage. This malfunction affects the complex mechanisms of mitophagy, biosynthesis, transport, fusion, and fission in the mitochondria. The main aspect of PD pathology is mitochondrial fragmentation, which is greatly aided by the aggregation of α-Synuclein (α-Syn) within the mitochondria. The proteins parkin and PINK1, which are essential for the management of damaged mitochondria and result in impaired mitophagy and neurodegeneration, are encoded by mutations in the PRKN and PINK1 genes, respectively. Within the mitochondria, Ca2+ inflow and efflux are selectively controlled and balanced by ligand-gated glutamate receptors such as N-methyl-D-aspartate receptors (NMDAR) and voltage-dependent ion channels. For the generation of ATP, neurons mostly depend on oxidative phosphorylation within the mitochondria and the activity-dependent control of cellular energy metabolism is ensured by mitochondrial Ca2+ absorption. Even slight modifications to Ca2+ homeostasis may have unfavorable effects and disrupt the physiological neuronal activity [39]. Specifically, two associated genes of PD that influence mitochondrial Ca2+ influx are PINK1 and PRKN. Using the Na+/Ca2+ exchanger, PINK1 regulates the release of Ca2+ from mitochondria, whereas PRKN opens voltage-dependent ion channels to facilitate Ca2+ diffusion through the outer membrane of the mitochondria. Therefore, reactive oxygen species (ROS) are produced as a result of mitochondrial Ca2+ excess brought on by PINK1 deficiency, which subsequently makes neurons susceptible to death.

### 2.4. Role of alpha-synuclein

The protein known as α-synuclein, or α-syn, is essential for maintaining synapses. It specifically affects the dopamine vesicle structural size, the location of DATs, and the synthesis of dopamine. This protein is found in many different parts of the brain, including the SN, hippocampus, neocortex, hypothalamus, and cerebellum [27]. Although α-syn is advantageous for physiological functions in its monomeric state, misfolding it and allowing it to aggregate into oligomers and fibers can result in neurodegenerative disorders. The precise process that initiates the change from monomeric α-syn to its aggregated forms is still unknown. Presently available pharmaceutical therapies for PD do not specifically target α-syn [28].

Since α-synuclein deposition also affects the peripheral nervous system (PNS) in the context of PD, there may be a connection between the pathogenic features of PD itself and the onset of peripheral neuropathies (PN). Studies have demonstrated that PN is more prevalent in individuals with PD than in healthy individuals within the same age group. PD has been associated with pathological alterations in the PNS, including α-synuclein deposits in the swallowing nerves and Lewy bodies in several nervous system locations, even in the early stages of the disease. These findings suggest that peripheral α-synuclein aggregates might contribute to the disease's spread to the central nervous system, where it results in the characteristic motor signs of the illness [29],[30]. Additionally, the discovery of α-synuclein clumps in the skin's unmyelinated fibers suggests that these markers may be used to diagnose PD early. Thus, comprehending how α-synuclein affects the peripheral and central neurological systems is essential for creating therapeutic strategies and diagnostic tools for PD and its associated conditions [23].

### 2.5. Role of Neuroinflammation

The DATs dynamic modulation in neurotransmission and homeostasis has been clarified by recent studies, emphasizing the DAT's function in dopamine signaling regulation and its significance in PD pathogenesis. Furthermore, new imaging methods have demonstrated how dopamine affects the brain's neuronal activity across the body, thus highlighting its effects on the motivation, behavior reinforcement, and movement. Knowing these pathways is essential to create tailored treatments that could change the course of PD and to enhance the patient outcomes. The part of neuroinflammation in PD may be a reaction to neuronal damage, as well as an initiating factor in the disease. For instance, the gut-brain axis has been connected to PD onset changes in the gut microbiome, which may set off an inflammatory cascade that impacts the central nervous system. Furthermore, the neurodegenerative process can be altered by genetic factors linked to immune response, which supports the increasing interest in anti-inflammatory drugs as possible therapies that could alter the course of the disease [31],[32]. A key role for neuroinflammation in the complex pathophysiology of PD, which impacts the course and beginning of the illness. To develop new therapeutic options targeted at either stopping or delaying the progression of PD, it is imperative to comprehend the underlying neuroinflammation mechanisms and how it interacts with other pathogenic processes [33].

**Figure 1.**
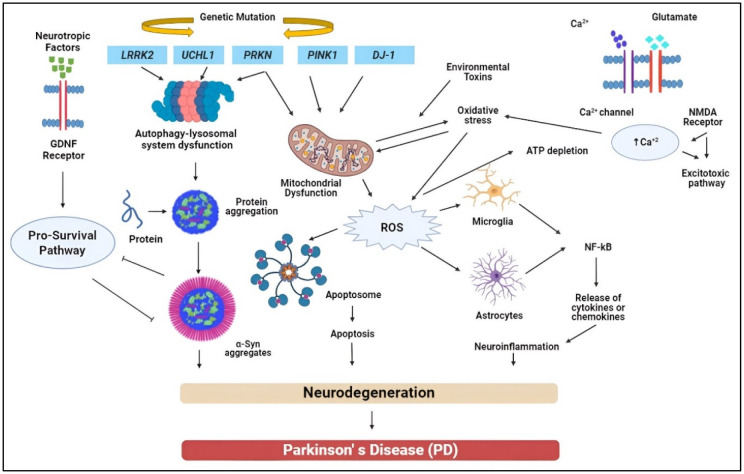
This figure represents the Parkinson's disease (PD) pathophysiology-related molecular pathways. In this, the common pathogenic pathways linked to PD are depicted, including altered intracellular Ca2+ homeostasis, reduced protein clearance, mitochondrial dysfunction, loss of trophic factors, and neuroinflammation. Solid arrows indicate signaling channels that have been strengthened, and blocked arrows indicate inhibited pathways. Leucine-rich repeat kinase 2 (LRRK2), parkin (PRKN), ubiquitin C-terminal hydrolase L1 (UCHL1), and phospholipase and tensin homolog-induced kinase 1 (PINK1) are all known as gland-derived neurotrophic factor, or GDNF. DJ-1: The acronyms for interleukin-1β, interleukin-6, and TNF-α are Junko-Daisuke-1, Alpha-synuclein (α-Syn), reactive oxygen species (ROS), tumor necrosis factor-alpha, IL-1β, IL-6, and N-methyl-D-aspartate (NMDA).

## 3. Current treatment of PD and their limitation

Neurodegeneration cannot be completely cured, and there are currently no drugs or treatments present on the market. Consequently, PD has no known cure at present. Only pharmacological interventions, such as dopamine substitutes and dopamine agonists, Monoamine oxidase -B (MAO-B) inhibitors, catechol-O-methyl-transferase (COMT) inhibitors, and decarboxylase enzyme modulators, can relieve the associated motor symptoms by restoring the striatal dopamine tone. The most efficient drugs presently utilized belong to the dopamine substitute category [34]. L-DOPA, which is considered the gold standard drug, is commonly used for clinical interventions. Levodopa and Carbidopa are combined for the best effect. The neurotransmitter tyrosine-hydroxylase (TH) can convert L-Tyrosine into L-DOPA, which is then further decarboxylated by DOPA to produce dopamine. Decarboxylase, MAO-A, and MAO-B, which are mostly found in the brain's glial cells, are primarily responsible for the metabolism of dopamine [35].

**Figure 2.**
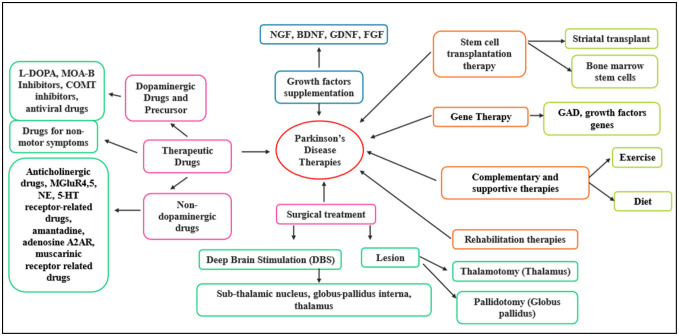
A variety of therapies are available to treat Parkinson's disease. Some treatments include medication modifications, surgery, gene and stem cell therapies, rehabilitative therapy, and other complementary and supportive therapies.

People often experience severe side effects after using levodopa medicine for a long period. Other symptom management treatments such as COMT inhibitors are unable to convert dopamine to inactive molecules. The goal of most novel therapeutics for PD involve restoring dopaminergic function in the striatum. However, these treatments don't stop the disease from progressing, nor do they address the non-motor symptoms that have the greatest impact on the patients' lives, such as cognitive impairment or stiffness in gait. Additionally, they do not address the non-dopamine-dependent complaints that may arise from PD. However, over time, their efficacy declines and their ability to reliably cure symptoms reduces. Additionally, L-DOPA is transferred to other brain regions, where it might cause cognitive decline and hallucinations. Furthermore, prolonged therapy may result in significant motor abnormalities and spontaneous movements; L-DOPA-induced dyskinesias (LID) is the term used to describe this condition. When administered in combination with a COMT inhibitor, L-DOPA and its peripheral turnover are reduced, thus extending the plasma half-life and enhancing the steady transport of L-DOPA to the brain. Deep Brain Stimulation (DBS) is a surgical procedure that is one of the additional PD therapy options. It has become a mainstay in the management of PD, particularly in patients who do not react well to medicine. During this procedure, electrodes are implanted in specific areas of the brain that generate electrical impulses to regulate abnormal impulses. Additionally, an implantable pulse generator (IPG) device, which is similar to a pacemaker, is a device that delivers electrical pulses to the brain by being inserted beneath the skin in the chest or abdomen. Surgery could be advantageous for patients who have LID. Despite significant unfavorable neuropsychiatric complications associated with DBS, it is a non-damaging therapy that is often employed for individuals who suffer from adverse effects caused by L-DOPA [36]–[38].

The current therapeutic agents that are used for the management of PD are described in the following table, alongside their mode of action adverse effects and the mode of effect in PD.

**Table 1.** This table provides a thorough summary of the medications used to treat Parkinson's disease, information on their modes of action, impact on the disease's symptoms, possible side effects, and links to reliable sources for more details.

**Mode of action**	**Agents**	**Adverse Effects**	**References**
Dopamine Replacement Agents	Levodopa Carbidopa	Dyskinesia, Sleep disturbances, Orthostatic Hypotension and Gastrointestinal issues.	[39]
Peripheral Decarboxylase Inhibitor	Benserazide	Nausea, Vomiting, Cardiac irregularities and Sleep disturbance.	[40]
Dopamine agonist	Bromocriptine Cabergoline Pramipexole Apomorphine Ropinirole	**Common** Nausea, vomiting, headaches, dizziness, hallucinations, **Serious** pulmonary fibrosis, Pleural effusion, Cardiac valvopathy, compulsive behaviour and orthostatic hypotension	[41]
MAO-B Inhibitors	Selegiline Rasagiline Safinamide	**Common** Nausea, dry mouth, lightheadedness, constipation **Serious** Hypertension, serotonin syndrome	[42]
COMT Inhibitors	Entacapone Tolcapone Opicapone	**Common** Nausea, diarrhoea, abdominal pain and urine discolouration **Serious** Dyskinesia, hallucination, and orthostatic hypotension.	[43]
NMDA Receptor Agonist	Amantadine	Nausea, dry mouth, headaches, dizziness, visual hallucinations, delusions, paranoia and livedo	[44]
Adenosine A2A Receptor Antagonist	Istradefylline	Dyskinesia, dizziness, constipation, nausea, hallucination, insomnia and uncontrolled muscle movement.	[45]
Cholinergic System Agents Anticholinergic Agents	Trihexyphenidyl Benztropine Biperiden Procyclidine	**Common** Dry mouth, blurred vision, constipation, urinary retention **Serious** Confusion, hallucination, memory impairment and tachycardia.	[46]
Antihistamine agents	Orphenadrine Promethazine	**Common** Dry mouth, dizziness, drowsiness, constipation **Serious** Hallucination, tachycardia, and respiratory depression.	[47]
Others and Miscellaneous Therapeis			

## 4. Advanced possible therapies

### 4.1. Surgical

Patients who take anti-PD drugs may experience several side effects, and their effectiveness is limited to a certain population of patients and for a short period. These drugs are unable to prevent a further loss of DA neurons. Physicians frequently resort to surgical procedures to alleviate motor symptoms when medicine is ineffective, especially in the later stages of PD. At the moment, PD is typically treated with two surgical procedures [48],[49].

#### 4.1.1. Deep Brain Stimulation Therapy

DBS is currently one of the most successful surgical therapies for severe PD, and has been utilized on a large number of patients worldwide. DBS involves implanting electrodes deep inside the brain, placing a pulse generator in the chest wall, and passing an electric current through a lead cable attached to the electrodes to stimulate the targeted deep brain region. New technologies have made it possible to treat PD in a personalized manner. In addition to choosing the DBS target and the stimulation parameters, DBS can be used on the target regions of the thalamus, globus pallidus interna (Gpi), or subthalamic nucleus (STN), with electrodes implanted in one or both hemispheres [50].

**Figure 3.**
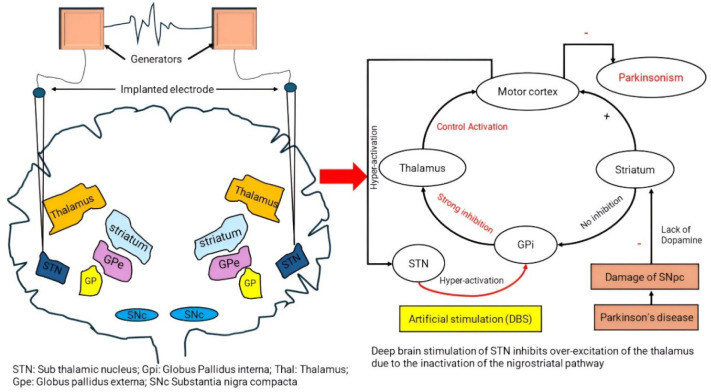
A schematic illustration illustrates the DBS procedure. In DBS, the thalamus, or in this case, the globus pallidus interna (Gpi) or STN, is stimulated by an implanted device made of batteries that produce electrical stimulation (much like a pacemaker). Stimulating the STN can enhance control over limb movement by engaging the Gpi, which can subsequently significantly block the thalamus (right-side circuitry) from activating the motor cortex.

The devices are implanted either under the collar bone or on both sides of the chest, with two batteries that produce precisely calibrated electrical currents to stimulate those deep brain locations in the DBS patients. These batteries produce electrical pulses, and they may be precisely tailored to meet the individual demands of PD patients. The implanted batteries can be inspected, changed, or recharged at intervals of three to five years. In addition to reducing the requirement for L-DOPA to treat dyskinesias, the DBS can help reduce many of the primary motor symptoms associated with PD. Additionally, a handheld device can be used to program the electrodes to turn on or off as needed [51].

#### 4.1.2. Surgical lesion (Pallidotomy or Thalamotomy)

The globus pallidus (GP) is responsible for regulating voluntary movements in the brain. It is a part of the basal ganglia and is closely connected to the thalamus and striatum. When a pallidotomy is performed, a portion of the GP is removed, thus disrupting synaptic connections with the thalamus or striatum. Therefore, because of damaged synaptic connections with the thalamus or striatum, PD patients have decreased bradykinesia, tremors, rigidity, and abnormal posture. After the surgical procedure, the amount of L-DOPA required by the PD patient may decrease, thus resulting in a reduction of drug-induced dystonia and dyskinesia. Thalamotomy is a similar procedure that involves the removal of the thalamus, which can disrupt the connection between the basal ganglia and the motor cortex, thus leading to a reduction in tremors and the restoration of neurotransmitter balance, including glutamate excitation. Thalamotomy is primarily used to manage tremors and is not effective in addressing dyskinesias, bradykinesias, and stiffness [52],[53].

### 4.2. Ablative surgery or lesioning

Lesioning or ablative surgeries (LS) involve the targeted destruction of specific brain tissue to disrupt maladaptive neural networks. Current techniques for LS include radiofrequency (RF) thermoablation, stereotactic radiosurgery (SRS), MRI-guided high-intensity focused ultrasound (HIFU), and laser interstitial thermal therapy (LITT). The first three methods are frequently employed to treat movement disorders [54]. As imaging and localization methods advance, LS continues to be a viable alternative for managing PD. LS is particularly considered for patients who either opt not to undergo DBS surgery, are unable to safely do so, or face challenges with regular follow-up programming visits [55]. Compared to DBS, LS is generally more cost-effective and reduces postoperative care requirements, as well as complications related to hardware. However, LS is not reversible, and any post-procedure adjustments are not possible without revision surgery. A significant drawback of LS is the increased risk of side effects with bilateral lesions, such as aphasia, dysarthria, dysphagia, and cognitive deficits, which occur in approximately 30–60% of bilateral thalamotomies and around 17% of bilateral pallidotomies, where patients may experience hypophonia, neuropsychological, and cognitive impairments [53],[56].

#### 4.2.1. Radiofrequency surgery by lesioning

Similar to DBS, RF lesioning involves the use of neuroimaging, a stereotactic headframe, and the insertion of an electrode into the brain, which is connected to a RF generator. The procedure is performed while the patient is awake, thus allowing for a test stimulation to confirm the correct target area. Then, a thermal lesion is created at the tip of the active electrode using an alternating current, and the electrode is removed once the lesioning is complete. RF lesioning is advantageous because it produces well-defined lesion borders with immediate effects, thus enabling the confirmation of symptom improvement during the surgery [54]. ViM thalamotomy, which is a procedure used to treat tremor-dominant PD, has demonstrated an immediate tremor reduction of 60% to 100% through RF ablation, with long-term benefits ranging from 57% to 90% lasting between 2 to 15 years. Common side effects of thalamotomy, such as ataxia, dysarthria, and sensory or motor deficits, are typically associated with perilesional edema. These side effects generally diminish over time, typically within a week to a month, although the duration can vary depending on the size of the lesion. Studies on unilateral pallidotomies using RF for PD have shown an average reduction of 30% in UPDRS III motor scores, with improvements in tremor, bradykinesia, rigidity, gait, and balance. Additionally, dyskinesia has been shown to improve by up to 90%. Adverse effects of pallidotomy may include visual field deficits, muscle weakness, and neuropsychological impairments, which are usually temporary and related to perilesional edemas of varying durations. The RF technique does carry some surgical risks, such as hemorrhages and infections [57],[58].

#### 4.2.2. Infusion of Levodopa and Carbidopa Intestinal Gel

A constant dose of a dopaminergic treatment is necessary to manage motor problems in patients with advanced PD. The Levodopa-Carbidopa Intestinal Gel (LCIG), which continually infuses levodopa into the jejunum, is an excellent choice. Using a tube that is introduced from the stomach into the jejunum, this technique maintains steady levels of levodopa in the blood, thereby reducing motor problems. Current research has validated the long-lasting effects of LCIG on lowering daily “off” time, with notable improvements continuing for long periods. Furthermore, LCIG has shown success in treating Freezing of Gait (FOG), which is resistant to other medications [59]. In addition to addressing motor symptoms, LCIG has been demonstrated to alleviate non-motor symptoms such as anxiety, depression, hallucinations, issues with impulse control, anxiety, and cognitive issues. However, compared to motor symptoms, the evidence for its benefits over non-motor symptoms is weaker, thus suggesting that more research is necessary. LCIG has advantages, but it is not without drawbacks. Pain, gastrointestinal distress, and device malfunction are frequent post-surgical problems that usually go away two weeks following the treatment. Prolonged use can cause neuropathy, gallbladder inflammation, and weight loss. It's critical to assess each patient's unique situation before LCIG is implemented, including any potential need for carer assistance. The process of identifying the best candidates for LCIG is continuous, and improving the patient outcomes heavily depends on the knowledge of a multidisciplinary medical team [60],[61].

#### 4.2.3. Focused Ultrasound Thermal Ablation

Focused Ultrasound (FUS) uses high-intensity ultrasound beams aimed at specific brain regions for thermal ablation. Guided by MRI and MR thermography, this technique, also known as MR-guided FUS (MRgFUS), allows precise targeting and real-time monitoring, thus reviving interest in lesioning procedures for movement disorders. The method involves using a transducer array in a helmet to direct ultrasonic waves through the skull to the brain. Benefits include no ionizing radiation, immediate results, precise lesion creation, and real-time MRI monitoring. However, drawbacks include patient claustrophobia in the MRI environment, longer procedure times, and the effectiveness being dependent on the skull thickness/density [62]. Currently, MRgFUS is FDA-approved for unilateral thalamotomies in essential tremor (ET) and tremor-dominant PD. Its application in pallidotomies and sub-thalamotomies is being researched. Studies have shown that MRgFUS thalamotomies improve UPDRS III motor scores by 30–60% in tremor-dominant PD, with similar results in pallidotomies. A recent study on MRgFUS sub-thalamotomy reported a 60.9% improvement in motor scores at 3 months. Another study that targeted the STN in 10 patients showed a 53% improvement at 6 months with minimal side effects. Over 6 months, 38 adverse events were recorded, with three directly related to STN lesioning, including choreic dyskinesia and speech disturbances; however, these symptoms significantly improved after medication adjustments [63].

## 5. Novel therapeutics

### 5.1. Gene therapies

PD gene therapy has advanced significantly over the past ten years. Advanced PD is not successfully treated with levodopa. Reduced levels of the enzyme aromatic amino acid decarboxylase (AADC), which changes L-DOPA into dopamine, are associated with an expansion of dopaminergic neuronal death [38].

The use of vectors such as lentivirus, adenovirus, and specifically recombinant adeno-associated virus (rAAV) is an emerging treatment method of PD known as viral vector-mediated gene delivery. Certain genes are carried by these vectors, which are injected either systemically or directly into the brain. When dopaminergic (DA) neurons are inside the host cell, they can stimulate gene expression that keeps them alive and stops them from degenerating, which raises dopamine levels. More recently, genes that encode neurotrophic factors, including GDNF, BDNF, and NGF, have been delivered using AAV2 vectors. These components are necessary for both the striatal rise of dopamine levels and the regeneration of DA neurons in the SN pars compacta (SNpc). For example, animals that received vectors that encoded GDNF have shown sustained increases in GDNF levels. An improved locomotor performance and a rise in DA terminals have been observed in primate models following the putamen injection of AAV2-GDNF. Additionally, it has been found that the introduction of the glutamic acid decarboxylase gene (GAD), which is required for GABA synthesis, by AAV vectors raised GABA levels in the subthalamic nucleus (STN). In PD brains, this aids in controlling the neuronal activity, which results in restored inhibitory signaling. According to clinical trials, AAV-GAD gene therapy in the STN is safe and well-received by individuals with advanced PD [64].

#### 5.1.1. Gene therapy by AADC-TH-GCH

The ability of the brain to manufacture neurotransmitters is compromised by a rare genetic disorder called aromatic L-amino acid decarboxylase (AADC) deficiency. Patients with AADC deficiency suffer from a lack of key neurotransmitters such as dopamine, serotonin, epinephrine, and norepinephrine, which leads to severe motor dysfunctions [65]. A novel gene therapy named Upstaza (eladocagene exuparvovec) has recently been recommended for approval in the European Union. Upstaza uses a modified virus to transport a functional AADC variant of the gene into nerve cells. Clinical studies have shown that patients with AADC deficiency who received Upstaza experienced improvements in their motor skills, including the ability to control their head and sit independently. Post-treatment rehabilitation is crucial to maximize the patient outcomes. Moreover, LV-GCH1-TH-AADC gene therapy has been explored for PD, with the aim of restoring key enzymes in the dopamine production pathway. Researchers have developed guidelines for a safe gene therapy application in AADC patients, and surgical techniques have been invented to directly deliver the gene therapy to the brain, thus enabling proper dopamine and serotonin production [66],[67].

#### 5.1.2. RNAi-based therapies

A potent method for suppressing genes linked to PD, including SNCA, PINK1, and parkin, is RNA interference (RNAi). Because of recent developments, molecular complexes that can penetrate cells and lower SNCA mRNA expression have been created. This decrease is noteworthy because it aids in preventing the neuronal death brought on by neurotoxins. Notably, utilizing RNA interference (RNAi) technology, researchers at the National Institutes of Health have discovered several genes that could be innovative therapeutic targets for the treatment of PD. Additionally, these findings may have relevance to other disorders associated with damage to the mitochondria. Furthermore, studies have demonstrated that RNAi can specifically lower the amounts of mutant α-synuclein RNA, which indicates that it may be useful as a PD2 therapy [68],[69].

#### 5.1.3. CSIRPR-Cas-9 gene editing therapy

New developments in the CRISPR-Cas9 technology push the limitations of the therapy of genes in PD, thus providing creative approaches to address the difficulties brought on by this neurodegenerative condition. CRISPR-Cas9 is a useful technique in the discovery of possible therapeutics because of its precision, which enables a targeted alteration and the correction of genetic abnormalities that contribute to PD. Current studies have emphasized the application of CRISPR/Cas9 in conjunction with stem cell therapies, thus highlighting its function in restoring gene abnormalities that cause neurodegenerative illnesses and creating animal models thereof. Moreover, the use of the CRISPR/Cas9 technology in PD research has received attention, with a focus on the development of innovative medical models relevant to PD, such as cellular models, small animal models, and big mammal models. Several gene editing techniques have been investigated concerning SNCA gene triplication, which leads to an excess of α-synuclein aggregation and plays a significant role in the pathogenesis of PD. For instance, a study reported using fluorescence-labelled markers to modify the mutant PD gene using CRISPR/Cas9 editing assisted by fluorescence-activated cell sorting (FACS). This method may be used to correct α-synuclein gene alterations linked to PD [70],[71],[72].

Additionally, the evolution of CRISPR-Cas9 and related gene editing systems has been detailed, with an emphasis on their application in PD research and the development of innovative medical models associated with the disease. These advancements demonstrate the ongoing efforts to understand and address the complex mechanisms which underlie PD. Towards the end of this section, it is important to note that the A53T mutation in the SNCA gene, although significant, is found in very few patients with PD. Consequently, treatments that target this specific mutation are limited in their applicability. The authors should provide this information to the reader to clarify the scope and limitations of the discussed techniques [73].

### 5.2. Immunization-based therapy

Potential treatments for PD have been investigated utilizing immunotherapeutic approaches, which center on removing superfluous alpha-synuclein (α-syn). Studies have shown that these treatments can improve behavioral problems in animal models and reduce α-syn aggregation. Since PD frequently results in neurotoxicity and cognitive deterioration due to an overactive immune system, immunotherapies may provide therapeutic benefits by reducing this hyperimmune response.

Novel strategies such as immuno-nanocarriers offer substitute treatment alternatives that may lessen PD symptoms by either inhibiting the production of Lewy bodies and α-syn oligomers, lowering oxidative stress and nitrogen compounds, or decreasing the release of cytokines that promote inflammation [74],[75].

Additionally, the application of monoclonal antibodies that target α-syn's C-terminal epitopes in immunotherapeutic approaches has demonstrated the potential in reversing behavioral irregularities in PD animal models and inhibiting aggregation of α-syn in neurons.

One important strategy to stop the spread of α-syn is to employ antibodies that recognize and neutralize the exogenous α-syn from cells, thus blocking it from reaching nearby cells. There are two types of immunotherapies against α-syn: passive immunization, which involves administering antibodies directly against α-syn, and active immunization, which induces the immune system to manufacture antibodies against α-syn. Preliminary human trials are currently being conducted, and the active and passive immunization strategies have confirmed neuroprotective effects in preclinical investigations. Recently developed therapeutic approaches, such as RNA-based tactics and antisense oligonucleotides, are being investigated to lower the synthesis of α-syn. Clinical studies for novel immunotherapeutic therapies, such as Cinpanemab (BIIB054), MEDI1341, AFFITOPE, and Prasinezumab (PRX002), which are presently undergoing clinical development and show promise for the management of PD, are among the most recent developments in the field. These cutting-edge methods highlight the continuous attempts to improve and hone PD treatment techniques, thus providing hope for better outcomes for patients [76],[77].

#### 5.2.1. Active immunization

The active immunization methods have the potential to provide long-term protection against the neurotoxic effects of protein misfolding by precisely targeting and maybe neutralizing harmful proteins linked to PD. Lately, there have been notable developments in this field regarding the development and testing of the following novel vaccine candidates:

UB-312: In a Phase 1 trial, this vaccine produced positive outcomes; in patients with PD1, treatment with UB-312 reduced the amount of harmful alpha-synuclein protein clumps by 20%. The goal of the vaccination was to mount an immune defense against these harmful protein aggregates; the results pointed to UB-312 as a potentially revolutionary PD treatment [74].

PV-1950R: A novel vaccine that focuses on three alpha-synuclein protein domains is currently advancing into the last stages of preclinical testing. By encouraging the production of antibodies that specifically target each of the three locations, this vaccination seeks to stop harmful clumps from forming [74]. For active immunization to be efficient in PD, it must overcome two major obstacles: modifying the immune tolerance of the host to the target antigens and ensuring a sufficient antibody passage across the blood-brain barrier (BBB). Overcoming these challenges will determine if vaccination techniques can change the course of PD and other neurodegenerative illnesses [9],[78].

#### 5.2.2. Passive immunization

Unlike immunization, passive immunotherapies take advantage of the epitope specificity of target antigens. Furthermore, the dosage and distribution of passive antibody therapies can be customized by utilizing the characteristics of the targeted antigen or the antibody itself. However, the need for repeated doses, perhaps over an extended length of time, is a disadvantage. Despite this, stable bioactive molecules with lengthy serum half-lives make up the bulk of antibodies utilized in passive immunotherapies. When safety and any adverse consequences are considered, the reversibility of the action is beneficial for passive dosing techniques [79].

Passive immunotherapies have recently made progress, which have resulted in the creation of effective vaccines; for example, the phase I clinical trial for the vaccine AFFITOPE PD03A, which was designed to contain a peptide that mimics α-syn, was recently completed. Another breakthrough includes a human monoclonal antibody called Prasinezumab (PRX002, Prothena), which targets the C-terminus of accumulating -syn. It has been revealed that during phase I clinical trials, the antibody can lower the free serum-syn by around 97% and has been well accepted for use. Furthermore, α-syn segments or α-syn-mimicking epitopes have been investigated in phase I trials as a means of inducing an aggressive autoimmune response against the protein. According to these trials, the therapies worked well when given subcutaneously. New α-syn targeted immunotherapies are still being developed; AstraZeneca and Takeda Pharmaceuticals are developing MEDI1341, which is a unique PD1 antibody. It is believed that antibodies against α-syn attach to extracellular α-syn and prevent it from passively immunizing new cells [80].

Prasinezumab is a type of antibody currently in the advanced stages of testing. Developed from the murine monoclonal antibody 9E4, this antibody is a humanized IgG1 monoclonal antibody. The 9E4 antibody targets epitopes close to the C-terminus of mouse α-syn and is known to identify amino acids 118–126 of α-syn. Together with other monoclonal antibodies under investigation, the goal of this strategy is to specifically target immune cells in PD and to open up new therapeutic options [81]. The administered antibodies through passive vaccination are thought to bind with extracellular α-syn and stop it from spreading [82].

The failure of the clinical trial for Prasinezumab in PD can be attributed to several interrelated factors. Primarily, the Phase 2 PASADENA trial did not demonstrate significant clinical improvements in the progression of motor and non-motor symptoms between the treatment and placebo groups, as measured by the MDS-UPDRS scores [83]. This lack of efficacy highlights the complexity of alpha-synuclein pathology, which exists in multiple forms, including monomers, oligomers, and fibrils. The trial targeted aggregated forms of alpha-synuclein; however, other forms may also play crucial roles in disease progression, complicating the therapeutic approach. Additionally, the timing of intervention is critical; while the trial involved early-stage Parkinson's patients, an even earlier intervention might be necessary to achieve a significant impact. The heterogeneity of PD, with its variations in symptoms, progression rates, and underlying pathology among patients, further complicates the ability to observe consistent treatment effects. Lastly, limitations in the study design, such as the patient selection criteria and the choice of endpoints, may have contributed to the trial's outcome. These factors collectively underscore the challenges in developing effective treatments for PD and highlights the need for more refined approaches in future studies [84],[85].

### 5.3. Treatment through Cellular Therapy

Cellular treatments have the potential to completely transform the way PD is treated because they offer a fresh method for either preserving or replacing dopaminergic neurons. This method uses cells that can be cultured in a lab and subsequently inserted into the brain, where they can aid in the restoration of normal brain function. Even though this method is still in its developing stages, preclinical research has indicated its potential, and it is currently being investigated as a possible PD treatment [86].

#### 5.3.1. Treatment with stem cells

Stem cells are the fundamental units of multicellular creatures because of their extraordinary capacity to self-renew and differentiate into several cell types. They are an essential source for the regeneration of tissues. They are often divided into two groups: somatic or adult stem cells, which are more limited and usually distinguish between distinct cell types related to the origin of tissue, and embryonic stem cells (ESCs), which come from embryos and have the capacity to develop into any cell type. The latest significant development in stem cell research is prompted pluripotent stem cells or iPSCs. iPSCs are derived from adult cells that have undergone genetic reprogramming to mimic embryonic stem cells. These cells are able to transform into any type of cell, including DA neurons, which are crucial in the development of PD. This reprogramming is accomplished through promoting the expression of particular pluripotency-associated transcription factors, such as Oct4, Sox2, Klf4, and Myc. Although iPSCs and ESCs have similar potentials for producing tissues for study and transplantation, ESCs raise additional ethical questions because their extraction results in the death of embryos. However, because they don't use embryos, iPSCs offer a more morally acceptable option [87]. Recently, promising advances have been made in the field of stem cell research for PD. Clinical trials are now being conducted to evaluate the efficacy and safety of stem cell infusion into the brain of PD patients to raise dopamine levels. For example, the STEM-PD clinical trial has advanced to evaluating greater dosages of cells following favorable safety reviews, with early indications of dopamine cell survival seen in the patient's brain PET imaging. Although iPSCs and ESCs have similar potentials for producing tissues for study and transplantation, ESCs raise additional ethical questions because their extraction results in the death of embryos. However, because they don't use embryos, iPSCs offer a more morally acceptable option [88].

These novel methods aim to treat the loss of SN neurons, which is the underlying cause of the motor symptoms of PD and may also deliver a more effective treatment plan for the condition. Stem cell therapies remain a glimmer of hope for changing the course of treatments for PD, and there is a requirement of further research and advancements for treating other neurological disorders [89].

### 5.4. Role of AI in the Management of PD

The role of Artificial intelligence (AI) in the management of PD is multifaced and AI has a great potential to overcome this medical emergency. AI has different aspects which can significantly improve the process of diagnosing and treating PD and attempt to play a key role in the management of both motor and non-motor symptoms. The usage of AI for the management of PD offers a cost-effective alternative to traditional methods. Studies have shown that AI can analyze gait patterns, speech signals, and EEG biomarkers for the disease progression adherence. Machine learning models and quantum computing integration show a high accuracy in the PD diagnosis; further research is needed to enhance the PD diagnosis and treatment [90].

#### 5.4.1. Early detection via AI

AI is a groundbreaking novel approach for the early detection of PD that leverages various innovative treatments. Researchers from the Massachusetts Institute of Technology developed an AI model that can estimate the occurrence of PD through measured nocturnal breathing patterns. An instrument is attached to a Wi-Fi router and includes the analysis of breathing signals by a neuronal network, either from a breathing belt or radio signal that arises during body sleep. This system gives information of the presence of PD and its severity [91]. Another important method to detect PD at the early stage includes the use of retinal scans. Scientists from Moorfield Eye Hospital and the UCL Institute of Ophthalmology employees utilized AI to analyze retinal markers from an optical coherence Tomography OCT scan. As a result, the pretty patient had a thinner layer in certain parts of the retina. Some other research has shown that blood biomarkers can also be detected through AI. This technique can predict the symptoms before 15 years; the noninvasive nature of blood tests makes this approach appealing for widespread use in under-detention [92].

#### 5.4.2. Personalized treatment

AI revolutionizes the management of PD by providing personalized treatment plans. AI can analyze patient data, identify patterns of optimized medication, guide rehabilitation and therapy, and continuously monitor patient symptoms and treatment effectiveness. This allows for more effective dosing schedules, reduced side effects, and improved patient outcomes. Additionally, AI helps in real-time feedback by adjusting therapy programs to individual needs and ensuring the most appropriate care at all times [93].

#### 5.4.3. Improving quality of life

AI improves the quality of life for partners and disease patients by managing non-motor symptoms. The AI system analyzes patient data to provide personalized recommendations for lifestyle adjustments, such as sleep hygiene and cognitive exercise. Moreover, AI-powered tools support rehabilitation and monitor movements. This integration of AI management enhances patient care and addresses individual challenges to improve the overall quality of life [92].

### 5.5. Drugs under clinical trial

The development of novel medications to treat PD is crucial for several key reasons. First, conventional treatment approaches mostly focus on the management of symptomatic relief rather than managing the disease progression. Therefore, developing novel therapeutics, especially those that target the key root causes of the disease, can lead to better outcomes to the symptom management, fewer side effects, and an enhanced quality of life of PD patients. Additionally, research for the development of novel medication advances the knowledge of the underlying mechanism of the disease and opens the door for personalized medicine strategies to tailor the specific subtype of PD or based on the individual unique genetic profile of PD patients. Furthermore, patients are given access to cutting-edge treatments, thereby taking part in these new medications' clinical trials advances medical knowledge. From an economic standpoint, the creation of novel medications can result in lower healthcare expenses related to managing illnesses, as well as an increased patient productivity and quality of life. In conclusion, the ongoing research and development of novel medications for PD has the potential to revolutionize available therapeutic approaches, advance the scientific understanding, and ultimately improve the quality of life for individuals who live with the illness. The major targets for PD drug development for management include the LRRK2, neurotrophic factors, alpha-synuclein, cell therapy, antioxidants, the microbiome, and GIT and dopamine precursors that will be the futuristic approaches to overcome PD [94].

**Figure 4.**
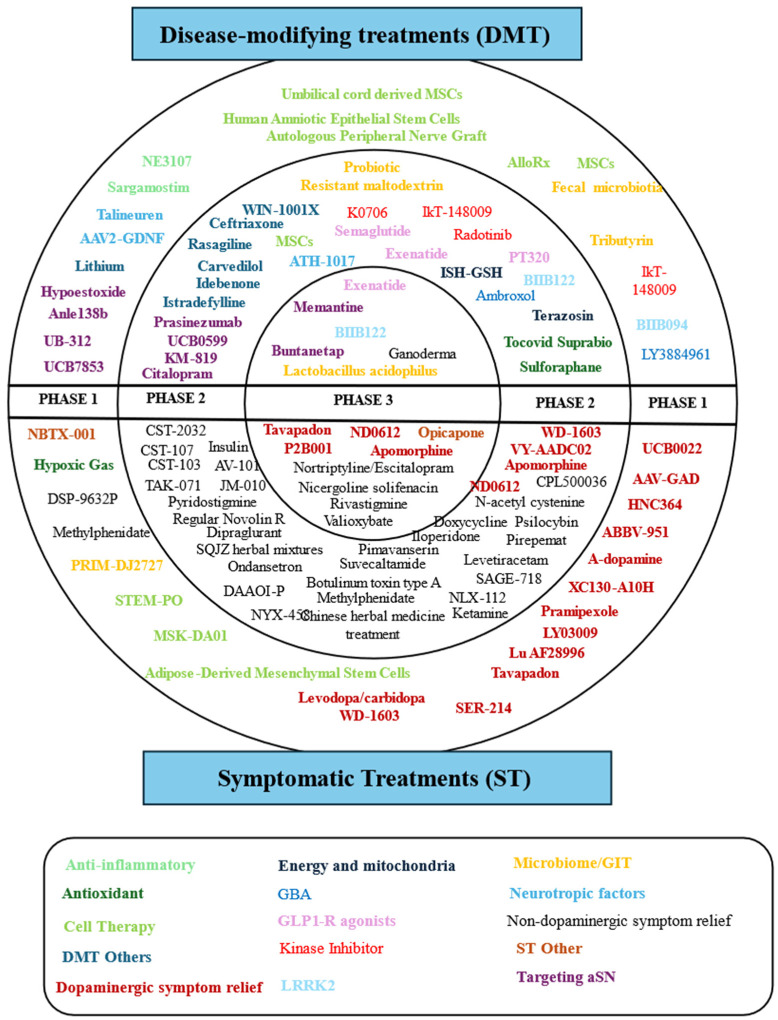
The drugs in this image are those that are undergoing clinical trials and are aimed at various approaches for the treatment of Parkinson's disease. All agents are registered and available on Clinicaltrail.gov.in.

### 5.6. Patented drugs for the management of PD

The research in recent years has shown good results in many areas and novel therapeutics are developed that have been patented. The table mentioned below describes with agent's name, patent ID, mode of action, and the issue and expiry dates for the granted patent.

**Table 2.** The mentioned table describes the agents that got patented for the treatment of PD recently from different countries all data registered on Intellectual Property India and Google patent search.

**Sr. No.**	**Patent ID/ Number**	**Drug Name**	**Mode of Action**	**Patent Granted**		**References**
				**Date of Issue**	**Date of expiry**	
1.	492170	Rutin and 5-HD	Reduce dopaminergic toxicity, mitochondrial function, integrity and bioenergetics	05/01/2024	05/01/2044	[95]
2.	382548	Rasagiline and Pramipexole (combination therapy)	Dopamine agonists	26/11/2021	26/11/41	[96]
3.	300584	Safinamide and its analogues	MAO-B inhibitor, Sodium channel blocker, calcium channel modulator	07/09/2018	07/09/2038	[97]
4.	254946	Trimethoprim	Antibiotic acts by inhibiting the DHFR	11/01/2013	11/01/2033	[98]
5.	CA2931082C	Nucleic acid products (e.g. primer)	Utilization of miRNAs from body fluids to detect and monitor PD	23/01/2024	17/11/2034	[99]
6.	US11466019B2	Triazolo[4,5-d] pyrimidine derivatives	Purine receptor (adenosine A2A antagonist)	11/10/2022	31/07/2029	[100]
7.	US20210161863A1	(+)-1-(3,4-chlorophenyl)-3-azabicyclo [3.1.0] hexane	Selective inhibitor of reuptake of monoamine neurotransmitters	01/05/2018	11/03/2034	[101]
8.	AU2019253834B2	Thienopyridine derivatives	Inhibitors of Acetyl CoA Carboxylase (ACC)	29/04/2021	09/11/2032	[102]
9.	US8524695B2	(6R)-4,5,6,7-tetrahydro-N6-propyl-2,6-benzothiazole-diamine	Dopamine receptors agonist	03/09/2013	03/01/2028	[103]
10.	JP6556146B2	Heterocyclic compounds	Inhibitory action, particularly targeting class II HDAC and HDAC6 inhibitory action	07/08/2019	25/08/2035	[104]
11.	ES2723876T3	New pyrazole derivatives	Inhibitory effect on phosphodiesterase 10 (PDE10)	03/09/2019	26/02/2034	[105]
12.	CN109125261B	Edaravone	Antioxidant and lipid peroxidation	30/11/2021	16/03/2036	[106]
13.	EP2592066B1	Amino carboxylic acid derivative	Reduction in no. of lymphocytes exhibits the immunoexpressed effect	03/12/2014	12/12/2025	[107]
14.	JP2021181448A	Brexpiprazole (OPC-34712)	Dopamine D2 Partial agonist, serotonin (5-HT2A) and Adrenaline A1 receptor antagonist	30/05/2023	-	[108]
15.	JP2018118914A	Combination of Febuxostat and Inosine	Enhancing ATP production in brain neurons.	17/11/2021	24/01/2037	[108]
16.	CA2806444C	Methyl Hydrogen Fumarate (MHF)	Immunomodulation inhibits inflammatory mediators and interaction with the TNF signalling pathways.	23/02/2016	19/08/2029	[109]
17.	CN102755310B	Combination of Levodopa and Amantadine	Improving motor function and fluctuations	15/06/2016	26/07/2032	[110]
18.	JP7443606B2	(4aR, 10aR)-1-n-propyl-1,2,3,4,4a,5,10,10a-octahydro-benzo[g]quinoline-6,7-diol	Dopamine agonist, provides dopaminergic innervation to the striatum and other brain regions, ultimately affecting the basal ganglia circuitry	05/03/2024	23/11/2038	[111]
19.	JP6251259B2	5-amino-5,6,7,8-tetrahydro quinoline-2-carboxylic acid	Soluble guanylate cyclase (sGC) activator, stimulating the biosynthesis of cGMP from GTP	20/12/2017	16/07/2033	[112]

## 6. Conclusion

Disease-modifying therapies are required for the better management of both motor and non-motor symptoms of PD, as the existing demography demonstrates that conventional therapies are neither capable enough to manage all the symptoms, nor are they able to modify the progression of the disease. The major thrust areas for future research should be in the areas of neuroplasticity and neuroprotective therapies. Neuroplasticity refers to the brain's capacity to reorganize itself by forming new neural connections throughout life. This concept is particularly relevant in PD as it may help in compensating the loss of DA neurons. Future research should prioritize identifying and enhancing mechanisms that promote neuroplasticity, including interventions such as physical exercise, cognitive training, and pharmacological agents that support synaptic plasticity. Regarding neuroprotective strategies, although PD is often diagnosed after the onset of motor symptoms, recent advances suggest that an early intervention could still offer significant benefits. Neuroprotective therapies aim to shield neurons from degeneration and slow disease progression. This can be pursued through various approaches, such as the use of antioxidants, anti-inflammatory agents, and drugs that inhibit apoptotic pathways. For instance, the use of drugs such as rasagiline and selegiline, which have demonstrated neuroprotective effects in preclinical studies, warrant further exploration.

Another important area is the CNS, as crossing the BBB necessitates advance drug delivery systems and medical devices with or without the use of artificial intelligence; one could be able to maintain the central dopaminergic tone by implementing such innovative idea/s. Similarly, the protection of neurons from degradation by the metabolic enzymes is a perspective module.

The future of PD therapeutic modules lies in multifaceted approaches that demand enhanced and precise diagnostic tools for a premature-deeper understanding of non-motor symptoms, personalized medications to tailor the individual needs, neuroprotective therapies to decelerate disease progression, comprehensive care modules that integrate psychological and social support, novel pharmacological agents that target the disease mechanism, and advanced therapeutic delivery systems for effective medication transportation. Such integrated novel perspective therapeutic strategies will not only be able to manage symptoms more effectively, but also improve the overall quality of life of PD patients. Novel symptomatic treatments for PD will be able to reduce motor complications from chronic L-DOPA exposure, thus avoiding the need for invasive treatments like DBS. The antagonist of adenosine A2A receptor and metabotropic glutamate receptor mGluR5 have shown promising results in trial studies, and such treatments will also enhance the dopaminergic tone and offer a better management of motor symptoms.
